# ﻿A new species of the pill millipede genus *Rhopalomeris* Verhoeff, 1906 (Diplopoda, Glomerida, Glomeridae) from Myanmar, and notes on *Rhopalomeriscarnifex* (Pocock, 1889)

**DOI:** 10.3897/zookeys.1215.130919

**Published:** 2024-10-16

**Authors:** Natdanai Likhitrakarn, Sergei I. Golovatch, Ruttapon Srisonchai, Parin Jirapatrasilp, Pichsinee Sapparojpattana, Ekgachai Jeratthitikul, Somsak Panha, Chirasak Sutcharit

**Affiliations:** 1 Program of Agriculture, Faculty of Agricultural Production, Maejo University, Chiang Mai 50290, Thailand Maejo University Chiang Mai Thailand; 2 Institute of Ecology and Evolution, Russian Academy of Sciences, Leninsky pr. 33, Moscow 119071, Russia Russian Academy of Sciences Moscow Russia; 3 Department of Biology, Faculty of Science, Khon Kaen University, Khon Kaen 40002, Thailand Khon Kaen University Khon Kaen Thailand; 4 Animal Systematics Research Unit, Department of Biology, Faculty of Science, Chulalongkorn University, Bangkok 10330, Thailand Chulalongkorn University Bangkok Thailand; 5 Animal Systematics and Molecular Ecology Laboratory, Department of Biology, Faculty of Science, Mahidol University, Bangkok 10400, Thailand Mahidol University Bangkok Thailand; 6 Academy of Science, The Royal Society of Thailand, Bangkok 10300, Thailand Academy of Science, The Royal Society of Thailand Bangkok Thailand

**Keywords:** Biodiversity, candy pill millipede, key, systematics, taxonomy

## Abstract

The taxonomy of the pill millipede genus *Rhopalomeris* Verhoeff, 1906, which is restricted to Indochina and currently comprises six described species, is refined and updated. An integrative taxonomic approach was employed that combines morphological examination with DNA barcoding using the cytochrome *c* oxidase subunit I (COI) gene for species identification and delineation. The first objective was to confirm the identity of *Rhopalomeriscarnifex* (Pocock, 1889), a charismatic species known as the “candy pill millipede” due to its vivid coloration, based on specimens collected near the type locality in Myanmar. The second objective was to describe a new species, *Rhopalomerisnigroflava* Likhitrakarn, **sp. nov.**, discovered in Linno Gu, Kayin State, Myanmar. This new species is distinguished by its small body size (5.1–9.7 mm long) and yellow body with contrasting brown to blackish markings on certain terga. In addition, the position of the telopod syncoxital lobe relative to the lateral syncoxite horns separates it from other *Rhopalomeris* species. The interspecific divergence between *R.nigroflava* Likhitrakarn, **sp. nov.** and other congeners ranges from 10.85% to 16.13%, based on uncorrected COI p-distances, while the intraspecific divergence was 0%–7.44%. A distribution map of and a revised identification key to all known species of *Rhopalomeris* are also provided.

## ﻿Introduction

**Figure 1.**
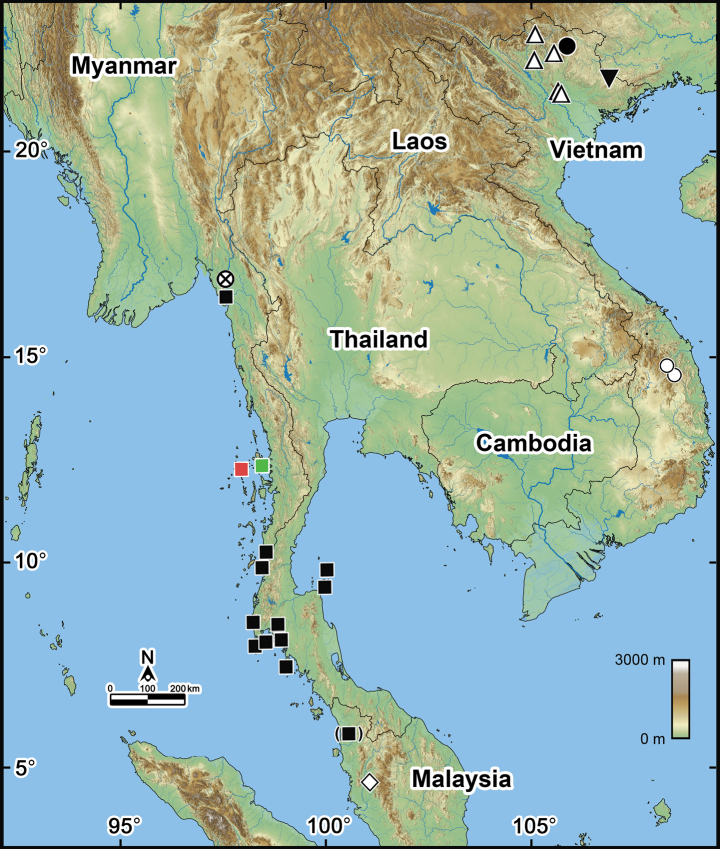
Distributions of all seven currently known *Rhopalomeris* species. Open triangles *Rhopalomerissauda* Nguyen, Sierwald & Marek, 2019; filled circle *Rhopalomerisnagao* Nguyen, Nguyen & Eguchi, 2021; inverted filled triangle *Rhopalomeristonkinensis* Silvestri, 1917; crossed circle *Rhopalomerisnigroflava* sp. nov.; filled squares *Rhopalomeriscarnifex* (Pocock, 1889); red square Elphinstone Island; green square Kala Island; open circle *Rhopalomerisvariegata* Golovatch, 2016; open diamond *Rhopalomerismonacha* Silvestri, 1917.

The Oriental genus *Rhopalomeris* Verhoeff, 1906 consists of only six species, all of which are restricted to Indochina (Golovatch et al. 2011; Golovatch 2017). The distribution range of this genus extends from the southern peninsular regions (Malaysia and Myanmar) to the North, encompassing Thailand and reaching as far as northern Vietnam (Fig. 1). All species except for *R.carnifex* (Pocock, 1889) show narrow distributions, while *R.carnifex* has been reported from a broader area that includes both Myanmar and Thailand (Fig. 1).

The genus *Rhopalomeris* belongs to the family Glomeridae. A total of 43 species of Glomeridae have so far been identified in Indochina and classified into six genera: *Annameris* Verhoeff, 1915 (two species), *Hyleoglomeris* Verhoeff, 1910 (23 species), *Hyperglomeris* Silvestri, 1917 (eight species), *Rhopalomeris* Verhoeff, 1906 (six species), *Peplomeris* Silvestri, 1917 and *Tonkinomeris* Nguyen, Sierwald & Marek, 2019 (one species each) (Likhitrakarn et al. 2014, 2023a, 2023b, 2024; Golovatch 2017; Golovatch and Semenyuk 2016; Nguyen et al. 2019, 2021).

Two unique morphological characters could be used to distinguish *Rhopalomeris* from the other glomerid genera: (1) antennomere 6 conspicuously enlarged, axe-shaped, exceeding the size of antennomeres 3–5 combined; (2) antennomere 7 also wide, topped by a disc-shaped antennomere 8 with numerous sensory cones, vs usually only four apical cones in other genera (except *Peplomeris*).

**Figure 2.**
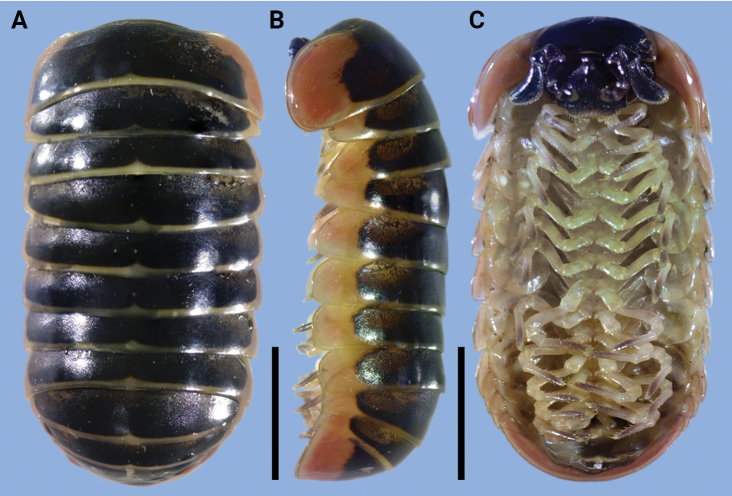
*Rhopalomeriscarnifex* (Pocock, 1889), ♂ specimen from Kala Island **A–C** dorsal, lateral, and ventral views. Scale bars: 5 mm.

A well-known *Rhopalomeris* species is *R.carnifex*, commonly referred to as the “candy pill millipede” or “rainbow candy pill millipede” because of its striking and vibrant patterns (Fig. 2). This characteristic has contributed to its popularity among exotic pet traders worldwide (https://www.reddit.com/r/millipedes/comments/xdrsr0/candy_pill_millipede/; https://undergroundreptiles.com/product/candy-pill-millipede/; https://www.exotic-pets.co.uk/candy-red-pill-bug.html; https://thespidershop.co.uk/product/rhopalomeris-carnifex/).

The present study employs an integrative taxonomic approach, combining both traditional morphological examinations and DNA barcodes derived from a fragment of the COI gene. The aims of this study are to re-evaluate the taxonomy of *R.carnifex* by examining specimens collected from Koh Kala, Tanintharyi Division, Myanmar, and to describe a new species discovered at Linno Gu, Kayin State, Myanmar. We also provide a comprehensive distribution map and a revised identification key to all species currently recognized in this genus.

## ﻿Material and methods

### ﻿Morphological studies

The new material was collected in Myanmar in 2015 and 2016 by SP and members of the Animal Systematics Research Unit, Chulalongkorn University, as well as by a French collecting team led by Louis Deharveng, of the Muséum national d’Histoire naturelle (MNHN), Paris, France. The locations of the collecting sites were recorded by GPS using a Garmin GPSMAP 60 CSx based on the WGS 84 datum, and all coordinates and elevations were double-checked using Google Earth. The collected specimens were euthanized using a two-step method following the AVMA Guidelines for the Euthanasia of Animals (AVMA 2013) and preserved in 90% (v/v) ethanol for morphological and molecular studies. After 24 h, the ethanol was replaced with 95% (v/v) ethanol to prevent defensive chemicals from interfering with DNA extraction.

The holotype and most paratypes are housed in the Museum of Zoology, Chulalongkorn University (**CUMZ**), Bangkok, Thailand. A few paratype duplicates have been deposited in the MNHN, Paris, France. The specimens were examined, measured, and photographed using a Nikon SMZ 745T trinocular stereo microscope equipped with a Canon EOS 5DS R digital SLR camera. Digital photographs were processed and modified using Adobe Photoshop CS5. The line drawings were based on photographs captured under a stereo microscope equipped with a digital SLR camera.

The terminology used to describe the morphological structures is consistent with that applied in the most recent publications (Golovatch et al. 2006; Golovatch 2017; Likhitrakarn et al. 2024).

In the catalogue sections, **D** stands for the original description and subsequent descriptive notes; **K** for the appearance in a key; **L** for the appearance in a species list; **M** for a mere mention; **MI** for molecular information; and **R** for new subsequent records.

### ﻿DNA extraction, PCR amplification, and sequencing

Total genomic DNA was extracted from the legs and part of the thoracic tissues using a DNA extraction kit for animal tissue (NucleoSpin Tissue Extraction Kit, Macherey-Nagel, Germany) following the standard procedure. The mitochondrial cytochrome *c* oxidase subunit I gene (COI: 660 bp) fragments were amplified using the primers LCO1490 and HCOoutout (Folmer et al. 1994; Schulmeister et al. 2002) or LoboF1 and LoboR1 (Lobo et al. 2013) using a T100™ thermal cycler (BIO-RAD) with a final volume 30 μL, DNA template 5 μL (15 μL EmeraldAmp GT PCR Master Mix, 1.5 μL each primer, 10 ng template DNA, and distilled water up to 20 μL total volume). Thermal cycling was performed at initial denaturation at 94 °C for 3 min, followed by 35 cycles of 94 °C for 30 s, annealing at 46 °C for 60 s in both primer sets, extension at 72 °C for 90 s, and final extension at 72 °C for 5 min. Amplification of the PCR products was confirmed by 1.5% (w/v) agarose gel electrophoresis before purification using MEGAquick-spinTM plus (Fragment DNA purification kit) and sequenced in both directions (forward and reverse) using an automated sequencer (ABI prism 3730XL).

All nucleotide sequences obtained in this study have been deposited in the GenBank Nucleotide sequence database under the accession numbers PQ219547–PQ219550. The collecting localities and GenBank accession numbers for each nominal species are listed in Table 1.

**Table 1.** List of the species used for molecular phylogenetic analyses and their relevant information. * = paratype.

Species	Voucher number	Locality	GenBank accession number COI	Reference
*Rhopalomeriscarnifex*	GLO016-1; GLO016-2	Myanmar	PQ219547; PQ219548	This study
*Rhopalomerisnigroflava* sp. nov.	GLO093-1*; GLO093-2*	Myanmar	PQ219549; PQ219550	This study
*Rhopalomerissauda*	IEBR-801; IEBR-706; IEBR-654; IEBR-533	Vietnam	MT749398; MT749400; MT749401; MT749404	Nguyen et al. 2021
*Rhopalomerisnagao*	IEBR-852; IEBR-854	Vietnam	MT749411; MT749392	Nguyen et al. 2021
*Hyleoglomeristongkerdae*	MUMNH-GLO071-1*	Thailand	P493218	Likhitrakarn et al. 2024
*Hyleoglomerisbomba*	MUMNH-GLO096-1*	Thailand	PP493219	Likhitrakarn et al. 2024
*Hyleoglomerissuwannakhuhensis*	MUMNH-GLO039*	Thailand	PP493220	Likhitrakarn et al. 2024
*Hyleoglomerisnigromaculata*	MUMNH-GLO019-1*; MUMNH-GLO019-2*; MUMNH-GLO019-3*	Thailand	PP493221; PP493222; PP493223	Likhitrakarn et al. 2024
*Hyleoglomerisdracosphaera*	MUMNH-GLO001-1*; MUMNH-GLO001-2*; MUMNH-GLO001-3*; MUMNH-GLO035-M2*; MUMNH-GLO035-F4*; MUMNH-GLO035-M1*; MUMNH-GLO035-F3*	Thailand	PP493224; PP493225; PP493226; PP493227; PP493228; PP493229; PP493230	Likhitrakarn et al. 2024
*Hyleoglomeriskrasoon*	MUMNH-GLO059*	Thailand	PP493231	Likhitrakarn et al. 2024
*Hyleoglomerishongkhraiensis*	MUMNH-GLO029-2*; MUMNH-GLO029-3*; MUMNH-GLO031-3*	Thailand	PP493232; PP493233; PP493234	Likhitrakarn et al. 2024
*Hyleoglomerisawaumi*	EG20210711-227-01; EG20210711-227-03; KS20210513-04; KS20210513-07	Japan	LC713407; LC713409; LC713416; LC713419	Kuroda et al. 2022b
*Hyleoglomerisinsularum*	EG20201213-09	Japan	LC713421	Kuroda et al. 2022b
*Hyleoglomerisjaponica*	MS20210617-01; MS20210617-02; MS20210617-03	Japan	LC713422; LC713423; LC713424	Kuroda et al. 2022b
*Hyleoglomerislucida*	EG20210718-240-01; MS20210426-11	Japan	LC713425; LC713426	Kuroda et al. 2022b
*Hyleoglomerissulcata*	MS20210521B-05	Japan	LC713428	Kuroda et al. 2022b
*Hyleoglomerisuenoi*	ST20211028	Japan	LC713429	Kuroda et al. 2022b
*Hyleoglomerishalang*	IEBR-Myr898P; IEBR-Myr926	Vietnam	ON704753; ON704754	Kuroda et al. 2022a
*Hyleoglomerislobus*	SVE-204; IEBR-653; IEBR-678	Vietnam	MT749391; MT749402; MT749406	Nguyen et al. 2021
*Hyperglomerisbicaudata*	CUMZ-GLO004*; CUMZ-GLO007*	Laos	OQ661871; OQ661872	Likhitrakarn et al. 2023a
*Hyperglomerisinkhavilayi*	CUMZ-GLO095/1*; CUMZ-GLO095/2*	Laos	OQ661873; OQ661874	Likhitrakarn et al. 2023a
*Hyperglomerissimplex*	IEBR-605; SVE-102	Vietnam	MT749403; MT749410	Nguyen et al. 2021
*Peplomerismagna*	IEBR-677; IEBR-656	Vietnam	MT749405; MT749408	Nguyen et al. 2021
*Tonkinomerisnapoensis*	IEBR-804b; IEBR-804a	Vietnam	MT749396; MT749397	Nguyen et al. 2021
*Trachysphaeracostata*	Tcost8-MK	Slovakia	KX467622	Mock et al. 2016
*Glomerismarginata*	ZFMK-TIS-18977; ZFMK-TIS-2517216	France	MG892125; MG892167	Reip and Wesener 2018
*Trachysphaeralobata*	ZFMK:MYR TW01	United; Kingdom	KJ408484	Wilbrandt et al. 2015
*Trachysphaeraschmidtii*	ZFMK:MYR BGIMyr16	Croatia	KJ408481	Wilbrandt et al. 2015
*Eupeyerimhoffiaarchimedis*	ZFMK:MYR1876	Italy	KP205574	Oeyen and Wesener 2015
*Sphaerobelumtruncatum*	CUMZ:2010.18	Thailand	JN885184	Wongthamwanich et al. 2012
*Zephronialaotica*	ZFMK:MYR3502	Laos	MK330977	Wesener 2019

### ﻿Phylogenetic analyses

The sequences were aligned using MEGA7 (Kumar et al. 2016). The final aligned dataset included 660 bp of the 61 COI sequences. The dataset included four sequences of *Rhopalomeris* that were newly obtained in this study and 57 sequences retrieved from the GenBank database, including all available sequences of *Rhopalomeris* species from Vietnam and other countries, and species of other genera in the Glomeridae (*Eupeyerimhoffia* Brölemann, 1913, *Glomeris* Latreille, 1802, *Hyperglomeris* Silvestri, 1917, *Peplomeris* Silvestri, 1917, *Hyleoglomeris* Verhoeff, 1910, *Tonkinomeris* Nguyen, Sierwald & Marek, 2019, and *Trachysphaera* Heller, 1858) (Table 1). *Sphaerobelum* Verhoeff, 1924 and *Zephronia* Gray, 1832 (order Sphaerotheriida, family Zephroniidae) were used as distant outgroups.

The best-fit substitution model was determined using PartitionFinder2 v. 2.3.4 (Lanfear et al. 2016) and used in subsequent phylogenetic analyses. The selected best-fit models for the three COI codon positions were SYM+I+G, GTR+I, and GTR+G, respectively. Phylogenetic relationships were reconstructed using two methods, maximum likelihood (ML) and Bayesian inference (BI) analysis, and through the online CIPRES Science Gateway (Miller et al. 2010). The ML analysis was calculated in IQ-TREE 2.2.2.7 (Minh et al. 2020) with 10,000 replicates of ultrafast bootstrap approximation to assess topology bootstrap support (BS). The BI analysis was estimated in MrBayes 3.2.7 (Ronquist et al. 2012) using the Markov chain Monte Carlo technique (MCMC). The BI trees were run for 10 million generations using a random starting tree. The resultant trees were sampled every 1,000 generations and the values were used to estimate the consensus tree topology, bipartition posterior probability (bpp), and branch lengths, after discarding the first 25% of the obtained trees as burn-in. The average effective sample size (ESS) from the MCMC analysis were > 1,800 for all parameters. The resulting tree was examined and edited using FigTree v. 1.4.3 (http://tree.bio.ed.ac.uk/software/figtree/, accessed on 28 February 2024). A clade was considered well supported if the ultrafast BS was ≥ 95% and Bayesian bipartition posterior probability was ≥ 0.95 (San Mauro and Agorreta 2010; Hoang et al. 2018).

Intraspecific genetic distances within taxa that contained more than one individual and interspecific genetic distances based on the COI sequences were also calculated using uncorrected p-distances, as implemented in MEGA7 (Kumar et al. 2016).

## ﻿Taxonomy

### ﻿Descriptions

**Family Glomeridae Leach, 1815**

#### ﻿Genus Rhopalomeris

Taxon classificationAnimalia

Verhoeff, 1906

1CD26737-F9B6-5527-883B-1C2B39261650

Rhopalomeris Verhoeff, 1906: 188 (D).
Rhopalomeris – Silvestri 1917: 140 (D); Jeekel 1971: 17 (L); Mauriès 1971: 435 (M); 2007: 243 (M); Hoffman 1980: 68 (L); Golovatch et al. 2011: 1 (D); Golovatch and Semenyuk 2016: 413 (D, K); Nguyen et al. 2019: 292 (D, K); 2021: 259 (D).

##### Diagnosis.

The genus *Rhopalomeris* could be recognized through numerous apical sensory cones on the antennal tip, and antennomere 6 being particularly enlarged and rather strongly curved. In addition, the posterior telopods are rather strongly enlarged and stout, supplied with both prefemoral and femoral trichosteles. The femur has a distinctive and particular distocaudal process. The body is relatively large, with adults ranging from 11 to 20 mm in length. The body coloration is variable, but often useful for species identification.

##### Type species.

*Glomeriscarnifex* Pocock, 1889, fixed under Art. 70.3 (ICZN 1999) in Golovatch et al. (2011), misidentified as *Rhopalomerisbicolor* (Wood, 1865) in the original designation by Verhoeff (1906).

##### Other species included.

*Rhopalomerismonacha* Silvestri, 1917; *R.tonkinensis* Silvestri, 1917; *R.variegata* Golovatch & Semenyuk, 2016; *R.sauda* Nguyen, Sierwald & Marek, 2019; and *R.nagao* Nguyen, Nguyen & Eguchi, 2021.

##### Remarks.

The genus *Rhopalomeris* was originally typified by Verhoeff (1906) through the designation of *Glomerisbicolor* Wood, 1865 as the type species. However, this designation was based on specimens from Salanga Island (presently known as Phuket Island, Thailand) housed in the Berlin Museum (currently Museum für Naturkunde Berlin; ZMB), and these specimens had been previously identified by F. Karsch as *G.bicolor*. Although the type locality of *G.bicolor* is in Hong Kong (Wood 1865), Verhoeff (1906) followed Karsch’s identification, and refrained to introduce a new name to those specimens.

Furthermore, Verhoeff (1906) compared the specimens from Phuket Island with *G.carnifex*, noting that several characteristics were similar to his specimens. He admitted that both might be two distinct species because of possible distinctions in telopods and the number of apical sensory cones on the antennae. Verhoeff also suggested to reclassify *G.carnifex* under the genus *Rhopalomeris*.

Subsequently, Silvestri (1917) examined relevant material probably housed in the Zoological Survey of India, (formerly the Indian Museum). He synonymized *G.bicolor* sensu Verhoeff (1906) with R.carnifexvar.pallida (Pocock, 1889) from Elphinstone Island, Mergui Archipelago, Myanmar, and redesignated *R.carnifex* from Tenasserim, Myanmar, as the type species.

Finally, Golovatch et al. (2011) studied the specimens of *G.bicolor* sensu Verhoeff (1906), from Salanga Island housed in the ZMB, and confirmed the identification of these specimens as *R.carnifex*. Golovatch et al. (2011) also synonymized the variety *pallida* with *R.carnifex* given the reason that the variety *pallida* was simply a color morph of *R.carnifex*, and validated that *R.carnifex* is the type species of *Rhopalomeris*, fixed under Art. 70.3 (ICZN 1999). Therefore, the millipede genus *Rhopalomeris* is currently known only from Myanmar, Thailand, Malaysia, and Vietnam, with a total of six nominal species involved (Fig. 1).

*Peplomeris* was originally described as a subgenus of *Rhopalomeris* (Silvestri, 1917). However, it was later raised to a genus level by Mauriès (1971), who assigned this genus to the tribe Haploglomerini, whereas *Rhopalomeris* belongs to the tribe Trachysphaerini (Mauriès, 1971). Nguyen et al. (2019) provided a comprehensive comparison among these two genera, highlighting key morphological differences among five Vietnamese glomerid genera. *Peplomeris* is characterized by simple, elongated telopods, the presence of a prefemoral trichostele, and a reduced to missing femoral trichostele. In contrast, *Rhopalomeris* has antennomere 6 that is unusually large, and trichosteles present in both prefemur and femur of the telopods. The antennae of *Rhopalomeris* also have numerous apical sensory cones like in *Peplomeris*.

#### ﻿ Rhopalomeris carnifex

Taxon classificationAnimalia

(Pocock, 1889)

8E00B92C-0188-5159-8340-4289A2E8B840

Figs 2 , 3

Glomeris carnifex Pocock, 1889: 290 (D). Type locality: Tenasserim.
Glomeris carnifex var. pallida Pocock, 1889: 290 (D). Type locality: Elphinstone Island.
Glomeris carnifex – Pocock 1890: 385 (R); Attems 1914: 138 (L); Hoffman 1980: 65 (M).
Glomeris carnifex var. pallida – Attems 1914: 138 (L); Silvestri 1917: 143 (D).
“Glomeris” bicolor [non Wood, 1865] – Verhoeff 1910: 241 (M); Silvestri 1917: 143 (M); Hoffman 1980: 65 (M).
Rhopalomeris carnifex – Silvestri 1917: 142 (D); Attems 1936: 194 (R); Enghoff 2005: 88 (R); Decker 2010: 24 (R); Golovatch et al. 2011: 6 (D); Golovatch and Semenyuk 2016: 411 (M, K); Likhitrakarn et al. 2017: 6 (L); Nguyen et al. 2019: 292 (M); 2021: 259 (M).
Rhopalomeris carnifex var. pallida – Silvestri 1917: 143 (D); Attems 1936: 194 (L).

##### Records in the literature.

Myanmar, south Tenasserim (Pocock 1889); Malwoon in Tenasserim (Pocock 1890); Elphinstone Island (Pocock 1889); Moulmein (Attems 1936); Taninthay Division, Tanintharyi Region, 9°56'20˝N, 98°32'22˝E, Thatay Kyun (= Pulo Ru, Ko Son) (Decker 2010). Thailand, Phuket Province, Salanga Island (= Phuket Island) (Verhoeff 1906; Enghoff 2005); Mueang Phuket District, Ko Siray, 7°53'7˝N, 98°26'14˝E, 20–50 m a.s.l. (Decker 2010); Krabi Province, Krabi District, Nai Chong (Enghoff 2005); Ao Luk, 8°10'54˝N, 98°50'30˝E, 70 m; Ban Khlong Jilat, 8°05'18˝N, 98°52'56˝E, 60 m a.s.l.; near Saengphet Cave, 8°9'46˝N, 98°53'12˝E, 80 m; Ko Lanta District, Ko Lanta Island, 80 m a.s.l.; Phang Nga Province, Ko Yao District, Ko Yao Noi, 8°9'53˝N, 98°37'20˝E, 150 m a.s.l.; Thap Put District, Had Lek Beach, 8°37'N, 98°13'E, 10 m a.s.l.; Khao Lak-Lamru National Park, 8°37'N, 98°14'E, 30–40 m; Surat Thani Province, Ko Samui District, Samui Island, Khao Phlu, 10–500 m a.s.l.; Nam Tok Na Muang Forest Park, 30 m a.s.l.; Ko Pha Ngan District, Phangan Island, Than Sadet-Ko Phangan National Park, 9°44'7˝N, 100°1'10˝E, 320 m a.s.l. (Decker 2010). Malaysia, neighboring the Malay Peninsula (Verhoeff 1906).

##### New material examined.

Myanmar – Tanintharyi Division • 2 ♂; Myeik, Kala Island; elev. 5 m a.s.l.; 12°29'38″N, 98°30'53″E; 5 Apr. 2016; C. Sutcharit, W. Siriwut, R. Srisonchai leg.; CUMZ-GLO016-1, 16-2.

##### Description.

Body length of unrolled specimens, 17.5–17.9 mm (♂), width 8.9–9.1 (♂).

Color faded after 15 years of preservation in alcohol (Fig. 2A–C): body blackish, with contrasting light yellow to yellow, broad to narrow bands at posterior edges of each of terga 2–11; axial stripe yellow, short, starting from behind caudal edge, not reaching 1/5–1/6 length of each tergite (Fig. 2A); in lateral view, terga 2–11 each with a large, reddish or carmine to red orange band each side, ~ 1/4–2/3 height of each tergum (Fig. 2B). Thoracic shield with a very large, lateral, reddish to red orange band each side at lateral edges, ~ 2/3–3/4 height of tergum in lateral view (Fig. 2A–C); anal shield (= pygidium) with a reddish to red-orange band at the lateral and posterior edges, ~ 2/3–3/4 height of tergum (Fig. 2B). Head, collum and antennae black to dark brown, only labrum and Tömösváry’s organ brownish (Fig. 2C), venter and legs brownish to pale yellowish (Fig. 2C).

**Figure 3.**
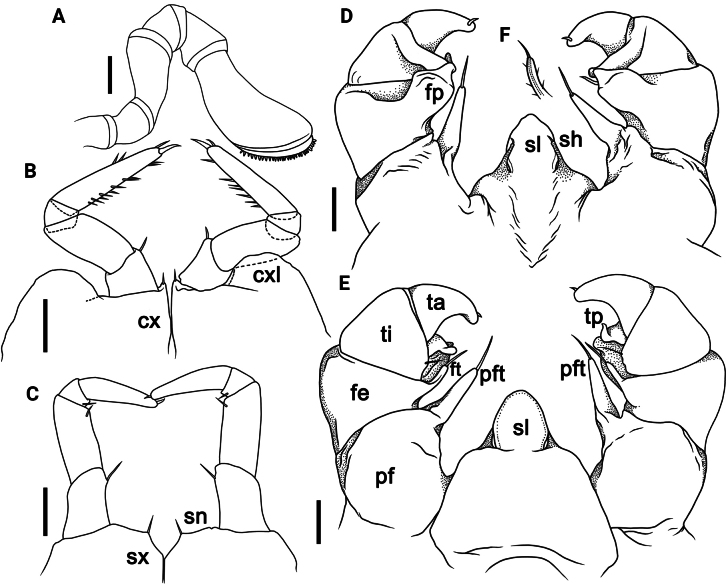
*Rhopalomeriscarnifex* (Pocock, 1889), ♂ specimen from Kala Island **A** antenna, anterior view **B** leg 17, anterior view **C** leg 18 anterior view **D, E** telopod, anterior and posterior views **F** tip of syncoxital lobes (without scaling). Scale bars: 0.5 mm. Abbreviations: cx coxa, cxl coxal lobe, fe femur, fp femoral process, ft femoral trichostele, pf prefemur, pft prefemoral trichostele of telopod, sh syncoxital horn of telopod, sl syncoxital lobe of telopod, sn syncoxite notch, sx syncoxite, ta tarsus, ti tibia, tp tibial process.

Labrum sparsely setose (Fig. 2C). Gnathochilarium with 2+2 palps subequal in length. Eyes blackish, with 8–11+1 ommatidia, cornea convex and translucent. Antennomere 6 long, ~ 2.3–2.5× as long as its height, dorsal margin strongly curved (Figs 2C, 3A). Disk of antennomere 7 beset with 55–62 small sensory cones apically (♂) (Figs 2C, 3A). Organ of Tömösváry typical, horseshoe-shaped, suboval, elongate, ~ 1.5–1.6× as long as broad.

Collum as usual, with two transverse striae. Thoracic shield with a small hyposchism field not projecting past rear tergal margin (Fig. 2B); 7–9 mostly superficial striae, only lower two or three lying in front of schism, one or two level to schism, remaining 2–4 behind schism, 6 and 7 complete striae, crossing the dorsum. Terga 3 and 4 relatively broadly rounded laterally (Fig. 2B). Following terga in front of pygidium faintly concave medially at caudal edge and with three or four striae starting above lateral edge, sometimes first and second stria fading away towards midway. Pygidium slightly concave medially at caudal edge.

♂ legs 17 (Fig. 3B) simple, rather strongly reduced, with a rather low to medium-sized, often rounded, coxal lobe and a 4-segmented telopodite. Tarsus with 4–10 strong median and 1–3 strong apical spines (Fig. 3B).

♂ legs 18 (Fig. 3C) simple, slightly reduced, without any evident outgrowths; syncoxite membranous, with a small, broad, arch-shaped syncoxite notch and a 4-segmented telopodite. Tarsus with a small, but strong apical spine.

Telopods (= ♂ legs 19) (Fig. 3D–F) with a rather large and roundly pentagonal syncoxite lobe, this being flanked by two short, spiniform, acuminate and setose syncoxite horns, the latter being evidently lower than syncoxite lobe (Fig. 3F). Telopodite 4-segmented, with a spine apically. Prefemur (Fig. 3D, E) subtrapeziform, with a conspicuous, elongated, robust, tuberculiform, distomesal prefemoral trichostele with a rounded tip, extending to about half or distal boundary of femur (Fig. 3D, E). Femur (Fig. 3D, E) subtrapeziform, with a stout, relatively short femoral trichostele in anterior view, extending apically to ~ 1/2–3/4 prefemoral trichostele, in posterior view with a rounded, slightly narrowed, subtrapeziform femoral process, this being strongly curved anterolaterally and gently tapering into an acuminate and pointed tip (Fig. 3D). Tibia stout, gently tapering distad and curved apicobasad towards femoral process, with an evident, distolateral tibial process, this being strongly curved mesad (Fig. 3E). Tarsus the smallest, subcylindrical, moderately sigmoid, strongly curved, narrowly rounded apically, with a robust and small terminal seta (Fig. 3D).

##### Remarks.

The taxonomic status of *R.carnifex* presents a challenge. Pocock (1889) originally described both *Glomeriscarnifex* and G.carnifexvar.pallida in the same paper. However, the original description of *G.carnifex* lacked details, focusing solely on body coloration and a vague collection locality (south Tenasserim, Myanmar). Pocock (1890) subsequently provided more information regarding the precise sampling locations, viz. south Tenasserim and Malwoon (= Maliwan, Kawthoung, Tanintharyi, Myanmar; Likhitrakarn et al. 2017).

In contrast, the description of G.carnifexvar.pallida from Elphinstone Island contained far more detail. Pocock (1889) provided information on the number of specimens (male and female individuals), body characteristics, the 18^th^ pair of legs and the telopod structure, all accompanied by clear illustrations. Notably, *G.carnifex* and G.carnifexvar.pallida differ only slightly in coloration, showing a central, longitudinal, carmine line and large, lateral, carmine spots on each tergite.

Subsequently, Silvestri (1917) provided a more detailed description, accompanied by comprehensive illustrations, while still treating *R.carnifex* and R.carnifexvar.pallida as two different taxa. He also treated *G.bicolor* sensu Verhoeff (1906) as a synonym with R.carnifexvar.pallida (Pocock, 1889). In a recent study, Golovatch et al. (2011) formally synonymized R.carnifexvar.pallida with *R.carnifex*.

However, our recently obtained specimens from Kala Island, Myanmar (Fig. 1, green square) closely resemble the original description of G.carnifexvar.pallida from Elphinstone Island (Fig. 1, red square) both in color pattern (Fig. 2) and morphological characters, especially the structure of their legs and telopod (Fig. 3) as described by Pocock (1889). These two geographically distant populations (ca 50 km apart) (Fig. 1) share these similarities, indicating that they probably belong to the same taxon. As R.carnifexvar.pallida is now synonymized under *R.carnifex* (Golovatch et al. 2011), we currently identify these specimens from Kala Island, Myanmar as *R.carnifex*.

Although there were previous reports of *R.carnifex* from several localities in southern Thailand, preliminary analyses of a number of *R.carnifex* specimens from this area reveal notable intraspecific variation in coloration, morphology, and molecular genetics, suggesting an occurrence of cryptic species (unpublished data). Therefore, a comprehensive redescription of newly retrieved male specimens from Kala Island, Myanmar in this study, comparing them with the original description of G.carnifexvar.pallida from the nearby Elphinstone Island, is essential before any taxonomic revisions of other Thai specimens can be proposed. Furthermore, the morphological redescription of *R.carnifex* above is thus based only on these Myanmarese specimens.

#### ﻿ Rhopalomeris monacha

Taxon classificationAnimalia

Silvestri, 1917

9187E385-EBE1-525B-801C-1762A546B354

Rhopalomeris (*s.s.*) monacha Silvestri, 1917: 143 (D).
Rhopalomeris monacha – Golovatch et al. 2011: 6 (M); Golovatch and Semenyuk 2016: 414 (M, K); Nguyen et al. 2019: 292 (M); 2021: 259 (M).

##### Remarks.

This species was described from Perak State, western Malaysia (Silvestri 1917). The species remains known only from a female holotype (Silvestri 1917). Endemic to Malaysia.

#### ﻿ Rhopalomeris tonkinensis

Taxon classificationAnimalia

Silvestri, 1917

25346F37-948C-516B-96AD-47EFC523A30A

Rhopalomeris (*s.s.*) tonkinensis Silvestri, 1917: 144 (D).
Rhopalomeris tonkinensis – Attems 1936: 194 (L); Golovatch 1983: 180 (L); Enghoff et al. 2004: 31 (L); Golovatch et al. 2011: 6 (M); Golovatch and Semenyuk 2016: 414 (M, K); Nguyen et al. 2019: 263 (L, M); 2021: 259 (M).

##### Remarks.

This species was described from Tonkin, Montes Mauson, 2,000–3,000 ft. a.s.l., Lang Son Province, northern Vietnam (Silvestri 1917). The species is likewise known only from a female holotype (Silvestri 1917). Endemic to Vietnam.

#### ﻿ Rhopalomeris variegata

Taxon classificationAnimalia

Golovatch & Semenyuk, 2016

47C0974C-D1B1-5BEB-A2A0-DE4E0D1C2C9D

Rhopalomeris variegata Golovatch & Semenyuk, 2016: 411 (D, K).
Rhopalomeris variegata – Golovatch 2017: 199 (D, R); Nguyen et al. 2019: 263 (L, M); 2021: 259 (M).

##### Remarks.

This species was described from Vietnam, Gia Lai Province, Kon Chu Rang Nature Reserve, 14°30'54″N, 108°32'47″E, ca 1,000 m a.s.l. (Golovatch and Semenyuk 2016) and later reported from Kon Tum Province, Kon Plong District, Bak Khe River, 14°43.450'N, 108°18.882'E, ca 1,000–1,260 m a.s.l. (Golovatch 2017). Endemic to Vietnam.

#### ﻿ Rhopalomeris sauda

Taxon classificationAnimalia

Nguyen, Sierwald & Marek, 2019

38CA835A-4D99-59F3-91D6-9062A63381E6

Rhopalomeris sauda Nguyen et al., 2019: 292 (D, K).
Rhopalomeris sauda – Nguyen et al. 2021: 259 (R, M, MI).

##### Remarks.

This species was described from Vietnam, Bac Kan Province, Ba Be National Park, 400–500 m a.s.l.; Vinh Phuc Province, Phuc Yen Town, Ngoc Thanh Commune, Me Linh Station for Biodiversity, 21.385°N, 105.7119°E; Tam Dao district, Tam Dao National Park, 21.460945°N, 105.647021°E (Nguyen et al. 2019); Tuyen Quang province, Cham Chu Nature Reserve; Ha Giang Province, Khau Ca Nature Reserve (Nguyen et al. 2021). Endemic to Vietnam.

#### ﻿ Rhopalomeris nagao

Taxon classificationAnimalia

Nguyen, Nguyen & Eguchi, 2021

BC3BA982-FF3D-532A-8DB9-82650F791A08

Rhopalomeris nagao Nguyen et al., 2021: 259 (D, K, MI).

##### Remark.

This species was described from Vietnam, Cao Bang Province, Pia Oac – Pia Den National Park, 22.5540°N, 105.8622°E, 850–1,600 m a.s.l. (Nguyen et al. 2021). Endemic to Vietnam.

#### ﻿ Rhopalomeris nigroflava

Taxon classificationAnimalia

Likhitrakarn sp. nov.

114E10A8-13AD-515D-AFB0-D15F62D30F2B

https://zoobank.org/6D8B6CCE-FED1-49C9-9E20-ADD3FD6AF618

Figs 4 , 5 , 6

##### Material examined.

***Holotype***: Myanmar – Kayin State • ♂; Linno Gu (Lateral small cave); 16°50'52.9"N, 097°36'37.7"E; 25 Nov. 2015; F. Bréhier leg.; MY15-13/01-CUMZ-GLO093. ***Paratypes***: Myanmar – Kayin State • 7 ♂♂ 5 ♀♀; same locality as holotype; MY15-13/01-CUMZ-GLO093) • 2 ♂♂ 2 ♀♀; same locality as holotype; MNHN-MY15-13/01.

##### Diagnosis.

**Figure 4.**
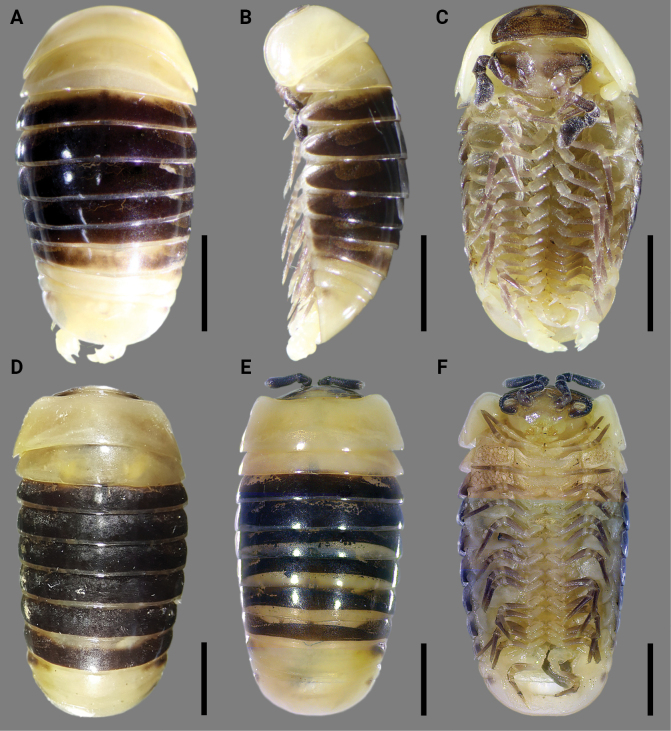
*Rhopalomerisnigroflava* sp. nov., **A–C** ♂ holotype in **A** dorsal **B** lateral and **C** ventral views **D–F** ♂ paratypes **D, E** dorsal and **F** ventral views. Scale bars: 0.2 mm.

Differs from other species of *Rhopalomeris* by the yellowish body with contrasting brown to blackish markings on terga 4–9 (Fig. 4A–F). Additionally, characterized by the smallest body sizes (5.1–9.7 mm in length and 2.6–4.7 mm in width), coupled with the telopod syncoxital lobe being slightly lower than lateral syncoxite horns. For further details, see key below.

##### Description.

Body length of unrolled holotype,7.3 mm, width 4.1 mm. Body length of unrolled paratypes, 5.6–9.3 mm (♂), 5.1–9.7 mm (♀), width 3.1–4.8 (♂), 2.6–4.7 mm (♀).

Color faded after nine years of preservation in alcohol (Fig. 4A–F): body yellowish to brown yellowish, with contrasting brown to blackish markings on terga 4–9 (Fig. 4A, B, D, E); lateral sides of terga 10, 11, and anal shield sometimes with a pair of small, faint, dark paramedian spots, these reaching neither caudal nor lateral edges (Fig. 4A, B, D, E); head, antennae and collum brown to dark brownish, only labrum, vertex and Tömösváry’s organ light brown; venter yellow brown to light yellowish; legs pale brown to brownish, with basal part of each podomere whitish (Fig. 4C, F).

**Figure 5.**
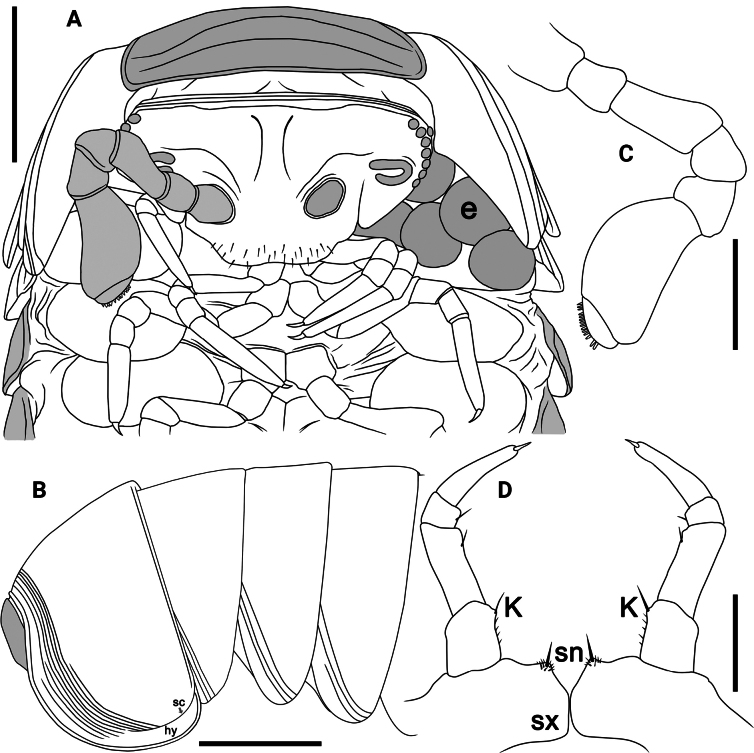
*Rhopalomerisnigroflava* sp. nov., ♂ holotype **A** head and anterior part of body, ventral view **B** thoracic shield, lateral view **C** left antenna, frontal view **D** leg 18, anterior view. Scale bars: 1 mm (**A, B**), 0.2 mm (**C**), 0.4 mm (**D**). Abbreviations: cxl coxal lobe, e eggs, hy hyposchism field, K caudomedial tubercle, sc schism, sn syncoxite notch, sx syncoxite.

Labrum sparsely setose (Figs 4C, F, 5A). Gnathochilarium with 2+2 palps subequal in length. Eyes blackish, with 6(7)+1 (♂) ommatidia (Fig. 5A), 6–(9)+1 ommatidia (♀), cornea convex and translucent. Antennomere 6 rather short, ~ 1.7–1.8× as long as its height, dorsal margin only slightly curved (Figs 4C, 5A, C). Disk of antennomere 7 beset with 22–28 small sensory cones apically (Fig. 5A, C), 16–26 small sensory cones apically (♀). Tömösváry’s organ typical, horseshoe-shaped, oblong-oval, elongate, ~ 1.6–1.7× as long as broad (Fig. 5A, C).

Collum as usual, with two transverse striae (Fig. 5A). Thoracic shield with a small hyposchism field not projecting past rear tergal margin (Fig. 5B). 7–9 mostly superficial striae, only lower 4 or 5 lying above schism, one level to schism, remaining 3 or 4 below schism, 6 or 7 complete, crossing the dorsum (Fig. 5B). Terga 3–7 rather broadly rounded laterally, with two or three striae starting above lateral edge, sometimes middle stria fading away mid-dorsally (Fig. 5B). Following terga in front of pygidium concave medially at caudal edge and with one or two striae starting above lateral edge. Male pygidium faintly concave medially at caudal edge (Fig. 4A, D, E).

**Figure 6.**
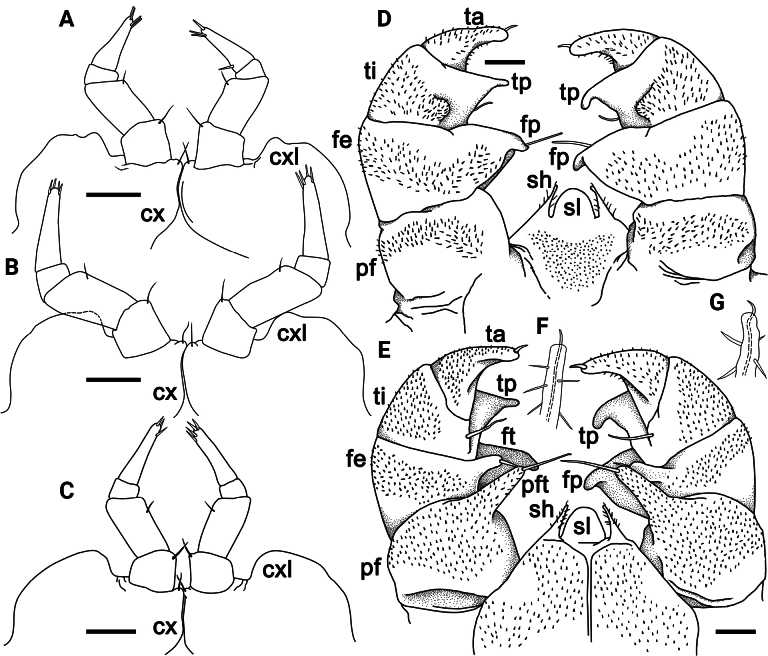
*Rhopalomerisnigroflava* sp. nov., **A, B** ♂ paratypes **C–F** ♂ holotype **A–C** leg 17, anterior view **D, E** telopod, posterior and anterior views, respectively **F, G** tip of syncoxital lobes (without scaling). Scale bars: 0.2 mm. Abbreviations: cx coxa, cxl coxal lobe, fe femur, fp femoral process, ft femoral trichostele, pf prefemur, pft prefemoral trichostele of telopod, sh syncoxital horn of telopod, sl syncoxital lobe of telopod, ta tarsus, ti tibia, tp tibial process.

♂ legs 17 (Fig. 6A–C) particularly strongly reduced, with a rather small to medium-sized, often irregularly rounded coxal lobe and a 4-segmented telopodite. Tarsus with 2–4 strong apical spines.

♂ legs 18 (Fig. 5D) rather strongly reduced, with a rounded ogival syncoxital notch and a 4-segmented telopodite. Femur with a small, setose, caudomedial tubercle near apex. Tarsus with a small apical spine.

Telopods (= ♂ legs 19) (Fig. 6D–G) with a small subtrapeziform, narrowly and roundly emarginated syncoxital lobe, this being flanked by two setose syncoxite horns, each of the latter higher than syncoxital lobe (Fig. 6D, E) and crowned by a subapical setoid filament (Fig. 6F, G). Telopodite 4-segmented. Prefemur (Fig. 6E) rectangular, with a conspicuous, elongated, robust, tuberculiform, distomesal prefemoral trichostele; in anterior view, with a rounded tip, extending to about half or distal boundary of femur (Fig. 6E). Femur (Fig. 6D, E) rectangular, with a prominent, stout, relatively short femoral trichostele in anterior view, extending apically to ~ 1/2–3/4 prefemoral trichostele, in posterior view with a rounded, subtriangular femoral process, this being curved anterolaterally and gently tapering into an acuminate rounded tip distally (Fig. 6D, E). Tibia stout, gently tapering distally and curved apicobasally towards femoral process, with a rather large, distolateral tibial process strongly curved mesad (Fig. 6D, E), with a strong anterior seta in anterior view (Fig. 6D) near base of tibial process. Tarsus the smallest, subcylindrical, moderately sigmoid, strongly curved, narrowly rounded apically, with a robust and small terminal seta (Fig. 6D, E).

##### Remarks.

It seems noteworthy that a female and two male (Fig. 5C) specimens were found guarding a clutch of eggs near its head, beneath the thoracic shield. This behavior deviates from the typical reproductive strategy so far known in the entire order Glomerida, where females deposit eggs in specialized clay chambers and leave them to develop independently (Thomas et al. 1970; Janssen 2013). This is the first instance of paternal brood care observed in Glomerida. Therefore, this newly discovered species presents fascinating traits worthy of a dedicated future study.

##### Etymology.

The specific epithet *nigroflava* is derived from the Latin *niger* meaning black and *flavus* meaning yellow, in reference to the dark bands on a yellowish dorsum, adjective in feminine gender.

### ﻿Key to known species of *Rhopalomeris*, based on adults, modified from Golovatch (2017)

**Table:** 

1	Thoracic shield yellowish or yellowish brown, contrasting to dark body background	**2**
–	Thoracic shield dark, variegated	**3**
2	Body larger, ~ 12 mm in length and 6 mm in width. Head, collum and thoracic shield light yellowish, body mostly blackish with a light, rather broad, axial stripe. Perak State, western Malaysia	***R.monacha***
–	Body the smallest, 5.1–9.7 mm in length and 2.6–4.7 mm in width. Body yellowish to yellowish brown-, contrasting to brown to blackish terga 4–9 (Fig. 4A, B, D, E). Head and collum brown to dark brownish (Fig. 5C, E, F). Kayin State, Myanmar	***R.nigroflava* sp. nov.**
3	Body large-sized, ~ 20 mm in length and 11 mm in width (♀). Dorsum: mostly blackish, not variegated. Mount Mau Son, Lang Son Province, northern Vietnam	***R.tonkinensis***
–	Body < 20 mm in length and 11 mm in width. Dorsum: with a varied color pattern or contrasting colors	**4**
4	Dorsum mostly dark, lateral edges of terga contrasting reddish or carmine. Telopod syncoxital lobe clearly higher than lateral syncoxite horns (Fig. 3D), each latter with a tiny filament on top (Fig. 3D, F). Southern Thailand and southern Myanmar (Fig. 1)	***R.carnifex ^1^***
–	Dorsum dark or light, sometimes variegated, lateral edges of terga neither reddish nor carmine. Telopod syncoxital lobe clearly shorter than lateral syncoxite horns, each latter without filament on top. Vietnam	**5**
5	Body almost entirely dark with contrasting four yellow lateral spots on each of terga 3–11. Telopods with a medially slightly concave syncoxite lobe. Prefemoral trichostele short, not extending to about half the distal boundary of femur. Femoral process (**fp**) long, narrow, erect, acute at tip. Tibial process short and lobuliform	***R.nagao***
–	Body color variegated. Telopods with a medially slightly convex syncoxite lobe. Prefemoral trichostele long, extending to about half or distal boundary of femur. Femoral process large, subtrapeziform, rounded at tip. Tibial process long and sigmoid mesally	**6**
6	Body light brown to blackish, with variegated, marbled, brown-yellow to yellowish markings. Body larger, 15–18 mm in length and 7–8 mm in width. Antennomere 6 slightly shorter, ~ 2.0× as long as high. Thoracic shield with 8–10 striae. Central Vietnam	***R.variegata***
–	Body blackish, with contrasting yellowish, lateral, oval bands on each of terga 2–11. Body smaller, 11 mm in length and 6 mm in width. Antennomere 6 longer, 2.5–3× as long as high. Thoracic shield with 2–3 striae. Northern Vietnam	***R.sauda***

### ﻿Phylogenetic analysis

**Figure 7.**
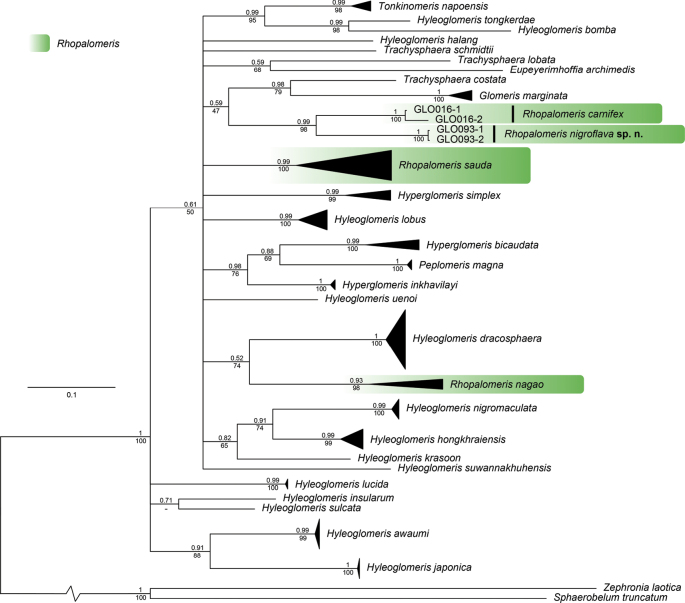
Bayesian inference tree (BI) of pill millipedes in the family Glomeridae based on 660 bp of COI gene. Clades of *Rhopalomeris* species in this study are highlighted in green. Numbers above branches indicate bipartition posterior probability (bpp) from Bayesian inference analysis (BI) and numbers below branches are Bootstrap Support (BS) values from the ML analysis.

The COI alignment (Table 1) was 660 bp in length and contained 61 individuals, including 29 taxa from the Glomeridae as ingroup and two taxa from the Zephroniidae as outgroup. The tree shows that all 29 pill millipede species from the eight genera of Glomeridae form a monophyletic group that is evidently separated from the outgroup, with strong support values (100% BS for ML and 1 bpp for BI) (Fig. 7). However, most of the relationships at the generic level among glomerid species still remained unresolved.

The COI tree revealed a sister relationship between *R.carnifex* and *R.nigroflava* sp. nov., forming a well-supported clade 98% BS for ML and 0.99 bpp for BI. However, all *Rhopalomeris* species, including *R.sauda* and *R.nagao*, were not retrieved together as monophyletic (Fig. 7).

The interspecific divergences based on COI uncorrected p-distance among the glomerid species in this study ranged from 9.74 to 19.87%, with an average of 14.87% (data not show). The interspecific divergences among *Rhopalomeris* species ranged from 10.85 to 16.13%, with an average of 13.32% (Table 2). This analysis also demonstrates that the intraspecific divergence for *Rhopalomerisnigroflava* sp. nov. is 0%, vs 1.75% for *R.carnifex*.

**Table 2.** Matrix of the average interspecific genetic divergence (uncorrected p-distance) for the 660 bp barcoding region of the COI gene between *Rhopalomeris* species.

Taxa	*Rhopalomerissauda*	*Rhopalomerisnagao*	*Rhopalomeriscarnifex*	*Rhopalomerisnigroflava* sp. nov.
*Rhopalomerissauda*	**0.0744**			
*Rhopalomerisnagao*	0.1289	**0.0521**		
*Rhopalomeriscarnifex*	0.1613	0.1397	**0.0175**	
*Rhopalomerisnigroflava* sp. nov.	0.1376	0.1235	0.1085	**0**

## ﻿Discussion and conclusions

Currently, the genus *Rhopalomeris* comprises seven species distributed across Vietnam (four species), Myanmar (two species), Thailand and Malaysia (one species each), with a notable absence of documented sympatry of species. The distribution patterns (Fig. 1), particularly the higher species diversity in Vietnam compared to the neighboring countries, suggest still a high probability of discovering new *Rhopalomeris* species in Cambodia and southern Thailand through future surveys.

*Rhopalomeris* species occur at elevations ranging between 5 and 1,600 meters above sea-level. Apparently, most are narrow endemics restricted to their type localities and are only rare to be encountered. The exceptions are *R.sauda* which boasts a wider distribution of roughly 180 kilometers, and *R.carnifex* that demonstrates a remarkably extensive range exceeding 1,200 kilometers and stretching from southern Myanmar through Thailand to northern Malaysia (Fig. 1).

It seems noteworthy that our preliminary surveys of millipede diversity in southern Thailand have yielded a high level of variation in the shape and coloration of *R.carnifex*, and high genetic diversity, suggesting a high-level intraspecific variation or the occurrence of cryptic species (unpublished data). This observation underscores the need for further research to comprehensively understand the extent of this variation, ultimately paving the way for future studies to definitively identify the *R.carnifex* complex.

The interspecific divergence based on COI uncorrected p-distance among the *Rhopalomeris* species in this study ranged between 10.85–16.13%, aligning with previous findings for European *Glomeris* species (11.5–17.1%; Wesener 2015), Vietnamese glomeridan genera (13–15.8%; Nguyen et al. 2021), *Hyperglomeris* species (8.81–12.48%, Likhitrakarn et al. 2023a), and *Hyleoglomeris* species (9.12–16.92%; Likhitrakarn et al. 2024).

This consistency suggests that COI proves effective in identifying species-level differentiation within Glomeridae. Even such species as *R.carnifex* and *R.nigroflava* sp. nov. that are very closely related and form a well-supported clade show a significant p-distance of 10.85%. This indicates that there can be considerable variability in the COI gene even among closely related glomerid species.

This study investigates the intraspecific COI divergence within Glomeridae millipedes. The low genetic intraspecific differences observed in *Rhopalomeriscarnifex* (1.75%) and the newly described *R.nigroflava* sp. nov. (0%) are consistent with previous reports on *Peplomerismagna* (0.2%; Nguyen et al. 2021), some *Hyleoglomeris* (0–1.19%; Likhitrakarn et al. 2024) and *Hyperglomeris* species (0.45–5.30%; Likhitrakarn et al. 2023a).

Analyzing the COI gene sequence is highly valuable in determining species boundaries and enabling precise classifications of glomerid species. Unsurprisingly, most recent taxonomic studies on millipedes frequently employ this technique to distinguish between taxa. Unfortunately, the phylogenetic relationships in this study appear insufficient to resolve genus-level relationships within the family, as shown in this study and others (Nguyen et al. 2019, 2021; Liu and Golovatch 2020; Likhitrakarn et al. 2023a, 2024). Subsequent research should include other genetic markers, such as 16S and 28S ribosomal RNA genes, as well as more advanced techniques, such as transcriptomic and phylogenomic data in clarifying phylogenetic relationships (Means et al. 2021; Benavides et al. 2023; Likhitrakarn et al. 2023a). Nevertheless, it is necessary to conduct these investigations combined with analyzing morphological, distributional, and ecological characteristics in order to obtain a more integrative comprehension of the evolutionary relationships among glomerid species, particularly regarding the intraspecific variation observed in the *R.carnifex* complex.

## Supplementary Material

XML Treatment for Rhopalomeris

XML Treatment for Rhopalomeris carnifex

XML Treatment for Rhopalomeris monacha

XML Treatment for Rhopalomeris tonkinensis

XML Treatment for Rhopalomeris variegata

XML Treatment for Rhopalomeris sauda

XML Treatment for Rhopalomeris nagao

XML Treatment for Rhopalomeris nigroflava

## Footnotes

The current key does not take the variation of R. carnifex from southern Thailand into consideration, see Remarks under R. carnifex above.

## ﻿Additional information

### Author ORCIDs

Natdanai Likhitrakarn https://orcid.org/0000-0002-1306-317X

Sergei I. Golovatch https://orcid.org/0000-0001-7159-5484

Ruttapon Srisonchai https://orcid.org/0000-0002-7142-0999

Parin Jirapatrasilp https://orcid.org/0000-0002-5591-6724

Pichsinee Sapparojpattana https://orcid.org/0009-0005-9134-4910

Ekgachai Jeratthitikul https://orcid.org/0000-0002-3477-9548

Somsak Panha https://orcid.org/0000-0002-4431-2458

Chirasak Sutcharit https://orcid.org/0000-0001-7670-9540

### Data availability

All of the data that support the findings of this study are available in the main text.
